# Bioinformatic and Functional Characterization of Hsp70s in Myxococcus xanthus

**DOI:** 10.1128/mSphere.00305-21

**Published:** 2021-05-19

**Authors:** Zhuo Pan, Zheng Zhang, Li Zhuo, Tian-yu Wan, Yue-zhong Li

**Affiliations:** a State Key Laboratory of Microbial Technology, Institute of Microbial Technology, Shandong University, Qingdao, People’s Republic of China; University of Wisconsin-Madison

**Keywords:** DnaK proteins, Hsp70 superfamily, *Myxococcus xanthus*, social behavior, stress response, transcription

## Abstract

Hsp70 proteins are among the most ubiquitous chaperones and play important roles in maintaining proteostasis and resisting environmental stress. Multiple copies of Hsp70s are widely present in eukaryotic cells with redundant and divergent functions, but they have been less well investigated in prokaryotes. Myxococcus xanthus DK1622 is annotated as having many *hsp70* genes. In this study, we performed a bioinformatic analysis of Hsp70 proteins and investigated the functions of six *hsp70* genes in DK1622, including two genes that encode proteins with the conserved PRK00290 domain (MXAN_3192 and MXAN_6671) and four genes that encode proteins with the cl35085 or cd10170 domain. We found that only MXAN_3192 is essential for cell survival and heat shock induction. MXAN_3192, compared with the other *hsp70* genes, has a high transcriptional level, far exceeding that of any other *hsp70* gene, which, however, is not the reason for its essentiality. Deletion of MXAN_6671 (*sglK*) led to multiple deficiencies in development, social motility, and oxidative resistance, while deletion of each of the other four *hsp70* genes decreased sporulation and oxidative resistance. MXAN_3192 or *sglK*, but not the other genes, restored the growth deficiency of the E. coli *dnaK* mutant. Our results demonstrated that the PRK00290 proteins play a central role in the complex cellular functions of M. xanthus, while the other diverse Hsp70 superfamily homologues probably evolved as helpers with some unknown specific functions.

**IMPORTANCE** Hsp70 proteins are highly conserved chaperones that occur in all kingdoms of life. Multiple copies of Hsp70s are often present in genome-sequenced prokaryotes, especially taxa with complex life cycles, such as myxobacteria. We investigated the functions of six *hsp70* genes in Myxococcus xanthus DK1622 and demonstrated that the two Hsp70 proteins with the PRK00290 domain play a central role in complex cellular functions in M. xanthus, while other Hsp70 proteins probably evolved as helpers with some unknown specific functions.

## INTRODUCTION

The 70-kDa heat shock proteins (Hsp70s) are highly conserved chaperone proteins that occur in all kingdoms of life. Under normal conditions, Hsp70s have a remarkable capacity to maintain proteostasis by assisting in protein folding, assembly, and degradation (1, 2). Hsp70s are typically induced in response to various environmental stresses, such as high temperature, oxidative damage, and antibiotic pressure, to help refold damaged proteins (3–5). In the *de novo* protein folding process, the canonical Hsp70 (DnaK in bacteria) system functions as a central hub in the chaperone network. It cooperates with upstream ribosome-associated factors in the folding of long nascent proteins and transfers complex and unresolvable nascent proteins to the downstream chaperonin (GroEL) or Hsp90 (HtpG) system (6, 7). Thus, Hsp70s play an important role in protein quality control.

**FIG 1:**
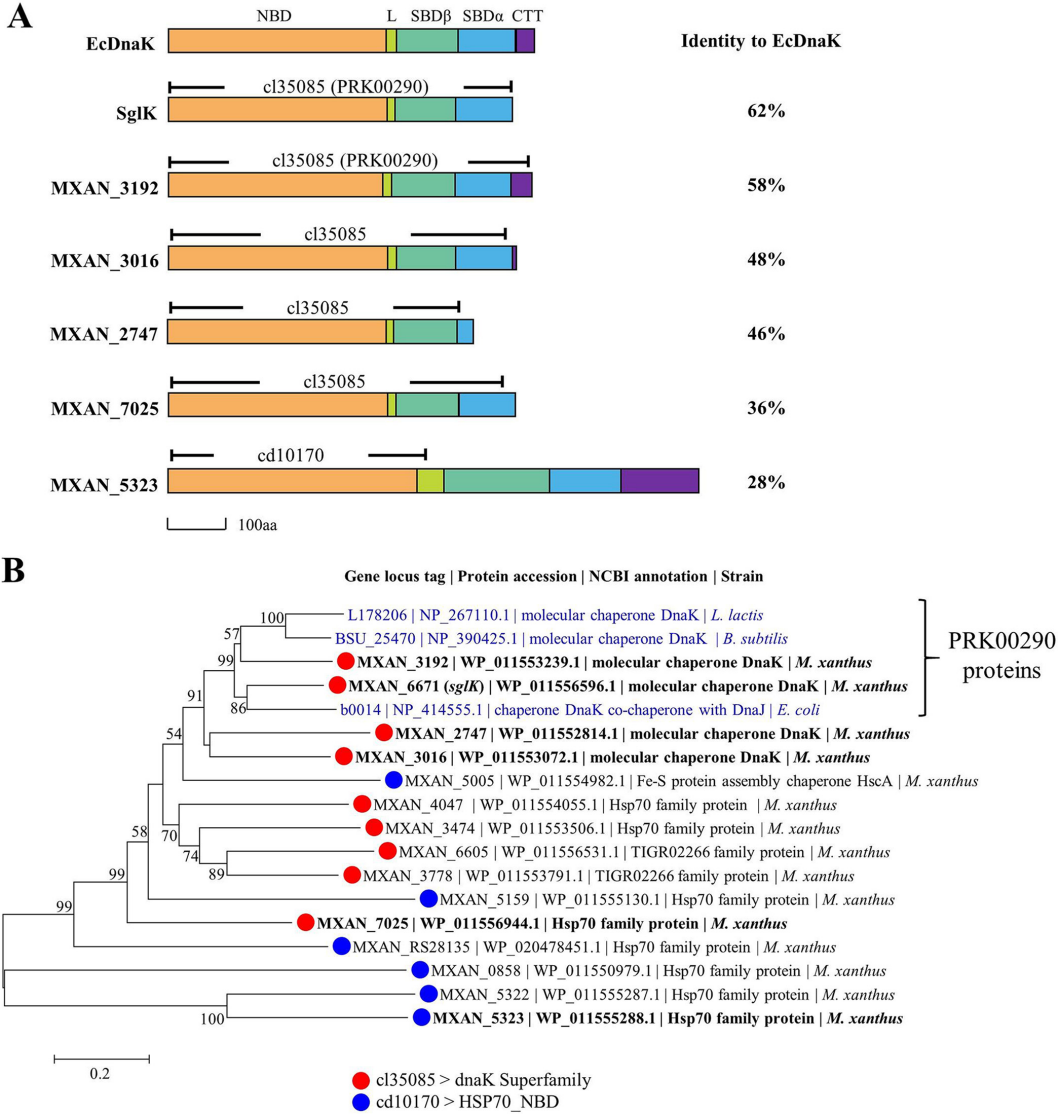
Phylogenetic characteristics of myxobacterial Hsp70s. (A) Overall architecture of EcDnaK and the six selected Hsp70s of M. xanthus. Information on corresponding functional domains and conserved domains is included. EcDnaK, DnaK from E. coli; NBD, nucleotide binding domain; L, linker; SBD, substrate binding domain; CTT, C-terminal tail. (B) Phylogenetic analysis of the 15 Hsp70 proteins from M. xanthus DK1622. The typical DnaK proteins (in blue) from E. coli, B. subtilis, and L. lactis were employed for comparison. Bars indicate a phylogenetic distance of 0.2.

An Hsp70 protein has two major functional regions: the N-terminal nucleotide binding domain (NBD) to bind and hydrolyze ATP and the C-terminal substrate binding domain (SBD) to offer a peptide substrate binding site. SBD is further divided into two subdomains: SBDβ directly binds to a peptide substrate, and SBDα functions as a lid to cover the substrate at the binding site (7, 8) (refer to DnaK of Escherichia coli in Fig. 1A). In addition, there is normally an unstructured C-terminal tail (CTT) preceding the lid domain with an unclear function (9). In the NCBI conserved domain database, a protein containing the cl35085 or cd10170 superfamily domain is annotated as an Hsp70 family protein. The cl35085 domain covers the NBD to SBD functional regions, while the cd10170 domain focuses only on the NBD region (9–11). Notably, the cl35085 superfamily presently contains a single member, the protein with the conserved PRK00290 domain, but there are many cl35085 proteins beyond the PRK00290 member in this superfamily. The proteins with the PRK00290 domain are usually characterized as canonical Hsp70 proteins (DnaK proteins).

It is well known that most eukaryotic cells have more than one kind of Hsp70, which is usually functionally redundant and diversified (12–14). For example, Saccharomyces cerevisiae contains 11 Hsp70s (15), six of which are localized in the cytosol (SSA1-4 and SSB1-2). Double deletion of *ssa1* and *ssa2* or *ssb1* and *ssb2* leads to a heat-sensitive or cold-sensitive phenotype, and the heat-sensitive phenotype can be compensated for by increasing the expression level of SSA3 rather than that of the SSB proteins (15). Similar functional redundancy and diversification of Hsp70s were also observed in human cells (16). In prokaryotes, the well-studied DnaK homologues are HscA and HscC of Escherichia coli; HscA is involved in the assembly of [Fe-S] clusters, and HscC protects chemically denatured substrates from aggregation, but neither is induced by heat shock (17). In the cyanobacterium *Synechococcus*, there are three *hsp70* genes, of which only *dnaK*2 is induced by heat shock, and it shows the highest sequence similarity with *dnaK* of E. coli (18, 19). Overall, many previous studies have focused on heat shock-inducible Hsp70 proteins in bacteria, but the multiple duplications and functional divergence of Hsp70s have been less well investigated.

Myxobacteria are a group of Gram-negative bacteria characterized by social behavior and a complex life cycle (20). The model strain of myxobacteria, Myxococcus xanthus DK1622, possesses a 9.14-Mb genome, which contains many duplicate genes, including different chaperones (21). Our previous studies investigated in detail the functional divergence, differentiation, and transcriptional regulation mechanisms of duplicate *groEL* genes in M. xanthus DK1622 (22–26). In contrast to the two duplicate *groEL* genes, DK1622 has been predicted to have 15 copies of *hsp70* in GenBank, of which only MXAN_6671 (*sglK*) and MXAN_3474 (*stkA*) have been explored. The transcription of these two genes is not induced by heat shock. StkA functions as a negative regulator of the biosynthesis of EPS (27), and SglK is involved in the production of extracellular fibrils and the processes of social motility (S-motility) and multicellular fruiting body development in M. xanthus DK1622 (28, 29). In this study, after a comprehensive bioinformatics analysis of the genes encoding Hsp70 proteins, we selected six *hsp70* genes, two encoding the PRK00290 proteins and four encoding the proteins containing the cl35085 (but not PRK00290) or cd10170 domain, to investigate their transcriptional and functional divergence. Our results demonstrated that the PRK00290 proteins play a central role in the complex cellular life cycle, while the other diverse Hsp70 superfamily homologues probably evolved as helpers with some unknown specific functions.

## RESULTS

### Existence of Hsp70s in bacteria, myxobacteria, and Myxococcus xanthus DK1622.

We screened for the presence of Hsp70s in 5,551 representative prokaryotic genomes (30) according to the annotation in the NCBI conserved domain database. We retrieved 6,147 genes encoding proteins in the cl35085 family, of which 5,815 contained the PRK00290 domain. An additional 3,477 genes containing the cd10170 domain were retrieved (see Data Set S1 in the supplemental material). The *hsp70* gene number in individual prokaryotic genomes ranged from 0 to 23; 99.1% of the genomes contained at least one *hsp70* gene, and 2,295 (41.3% of the total prokaryotic genomes) possessed two or more *hsp70* genes. The results suggested that the *hsp70* gene is often duplicated in prokaryotic cells, such as in myxobacteria. The gene encoding the PRK00290 protein was duplicated in 316 genomes, accounting for 5.7% of the total prokaryotic genomes. Notably, the proteins that belong to the cl35085 superfamily but are not PRK00290 proteins are considerably rare in bacteria, accounting for only 5% of the total cl35085 proteins (310 of 6,146) and 4.4% (242) of the total 5,551 genome-sequenced bacteria. Comparatively, all the myxobacteria possess these cl35085-but-not-PRK00290 proteins (Table S1A).

10.1128/mSphere.00305-21.6 DATA SET S1 Existence of the *hsp70* genes encoding the proteins with the PRK00290, cl35085, or cd10170 conserved domain. Download Data Set S1, XLSX file, 0.4 MB. Copyright © 2021 Pan et al. 2021 Pan et al. https://creativecommons.org/licenses/by/4.0/ This content is distributed under the terms of the Creative Commons Attribution 4.0 International license.

10.1128/mSphere.00305-21.8 TABLE S1 Occurrence of *hsp70* genes in (A) 38 sequenced myxobacterial genomes and (B) M. xanthus DK1622. Download Table S1, TIF file, 2.8 MB. Copyright © 2021 Pan et al. 2021 Pan et al. https://creativecommons.org/licenses/by/4.0/ This content is distributed under the terms of the Creative Commons Attribution 4.0 International license.

Myxobacteria, which are renowned for their complex life cycles, are a representative of the bacterial groups that contain many duplicate genes (21), including multiple copies of *hsp70*. The number of genes encoding proteins with cl35085 (PRK00290) and cd10170 domains varied from 3 to 10 (1 to 3) and 3 to 7, respectively, in 38 sequenced myxobacterial genomes (Table S1A). Thirty-four myxobacterial genomes possess two genes encoding the PRK00290 proteins, and the other four genomes have three copies or one copy. The genomes of Corallococcus carmarthensis DSM 108842 and Nannocystis exedens DSM 1461 each possess only one copy of the PRK00290 gene, while Minicystis rosea DSM 24000 and Vulgatibacter incomptus DSM 27710 both have three copies. We compared the whole-genome-based phylogenetic tree of 38 sequenced myxobacteria and the phylogenetic tree of the 76 PRK00290 proteins in these genomes (Fig. S1A and B). The results showed that the PRK00290 proteins were obviously separated into two subbranches, and the two subbranches each displayed a highly similar phylogenetic relationship with the myxobacterial phylogenetic tree. Notably, the missing PRK00290 proteins in *C. carmarthensis* DSM 108842 and *N. exedens* DSM 1461 are in group 1; the third PRK00290 proteins in *M. rosea* DSM 24000 and *V. incomptus* DSM 27710 are in group 2. Thus, group 2 PRK00290 proteins probably play a more essential role in myxobacteria.

10.1128/mSphere.00305-21.1 FIG S1 Phylogenetic and transcriptional characteristics of myxobacterial Hsp70 proteins. (A) Phylogenetic tree of the PRK00290 proteins from 38 myxobacteria. The topologies of group 1 and group 2 were highly similar to that of the phylogenomic tree based on the genome sequences (B). (B) Phylogenomic tree constructed using 38 sequenced myxobacterial genomes. (C) Transcriptional levels of the hsp70 genes in M. xanthus DK1622 under normal growth and heat shock conditions. FPKM, number of fragments per kilobase of transcript sequence per million base pairs sequenced. Download FIG S1, TIF file, 1.6 MB. Copyright © 2021 Pan et al. 2021 Pan et al. https://creativecommons.org/licenses/by/4.0/ This content is distributed under the terms of the Creative Commons Attribution 4.0 International license.

In M. xanthus DK1622, there are nine genes encoding the cl35085 proteins (including two genes for the PRK00290 proteins) and six genes encoding the cd10170 proteins (Table S1B). However, four genes were annotated by the NCBI database as encoding the molecular chaperone DnaK, including two proteins with the PRK00290 domain (MXAN_3192 and SglK) and two proteins with the cl35085 domain (MXAN_2747 and MXAN_3016). A phylogenetic analysis of the 15 Hsp70 proteins based on their amino acid sequences showed that the two PRK00290 proteins were located in the same subbranch as the typical DnaK proteins from E. coli, Bacillus subtilis, and Lactococcus lactis, while the cl35085 proteins were close but located in different subbranches from the typical DnaK proteins. Most of the cd10170 proteins were more distant from the typical DnaK proteins than the cl35085 proteins (Fig. 1B).

A typical Hsp70 is usually characterized by a high expression level and a positive heat shock response (4). Our transcriptomics data showed that, of the 15 *hsp70* family genes, MXAN_3192 exhibited a high transcriptional level (the number of fragments per kilobase per million reads [FPKM] of MXAN_3192 was 1,100, and the median FPKM of the total 7,348 genes in DK1622 was 31), while the transcription of all the other genes was comparatively low (Fig. S1C; the transcriptomics data are provided in Data Set S2). For example, the transcription of *sglK*, the second most highly expressed *hsp70* gene in DK1622, reached only approximately one-eighth of that of MXAN_3192. Otani et al. identified 18 major heat shock-induced proteins in M. xanthus (31), which included MXAN_3192 but not the other Hsp70 proteins. Consistent with this, we found that the transcription of MXAN_3192 was induced (∼6-fold) by heat shock, while the other *hsp70* genes, except MXAN_5005 and MXAN_5323, exhibited unchanged or decreased expression in response to heat shock.

10.1128/mSphere.00305-21.7 DATA SET S2 Transcriptomics data of M. xanthus DK1622 under normal and heat shock conditions. Download Data Set S2, XLSX file, 1 MB. Copyright © 2021 Pan et al. 2021 Pan et al. https://creativecommons.org/licenses/by/4.0/ This content is distributed under the terms of the Creative Commons Attribution 4.0 International license.

### MXAN_3192, but not other *hsp70* genes, is essential for the viability of M. xanthus DK1622 cells.

To analyze the functions of the diverse Hsp70 proteins in M. xanthus, we selected six Hsp70s (Fig. 1B; the six selected Hsp70 proteins are in bold). Four of the selected Hsp70 proteins, namely, MXAN_2747, MXAN_3016, MXAN_2747 (SglK) and MXAN_3192, were phylogenetically close to the typical DnaK proteins; they all possessed the cl35085 domain, but two (SglK and MXAN_3192) were PRK00290 proteins. These four Hsp70 proteins were all annotated by NCBI as “molecular chaperone DnaK.” The other two selected Hsp70 proteins, namely, MXAN_5323 and MXAN_7025, were cd10170 and cl35085 family proteins, respectively. In the phylogenetic tree, MXAN_5323 and MXAN_7025 were rather distant from the typical DnaK proteins among the cd10170 or cl35085 proteins (Fig. 1B). The six selected Hsp70 proteins varied significantly in amino acid identity from the typical DnaK proteins (Fig. S2) but had similar sequence architectures with some variations. The NBD of the six *Myxococcus* Hsp70s were highly conserved, while the SBD and CTT domains varied markedly: the CTT was absent in MXAN_2747, MXAN_3016, SglK, and MXAN_7025, and the SBDα of MXAN_2747 was much shorter than those of the other proteins. Comparatively, MXAN_5323 contained 925 amino acids, far exceeding the 638 amino acids of EcDnaK or the other five *Myxococcus* Hsp70s (Fig. 1A).

10.1128/mSphere.00305-21.2 FIG S2 Sequence alignment of six Hsp70s from M. xanthus DK1622. Three typical DnaK proteins were employed for comparison. EcDnaK, DnaK from E. coli (accession no. NP_414555.1). LlDnaK, DnaK from L. lactis (accession no. NP_267110.1). BsDnaK, DnaK from B. subtilis (accession no. NP_390425.1). The secondary structures are based on PDB entry 2KHO. Download FIG S2, TIF file, 2.1 MB. Copyright © 2021 Pan et al. 2021 Pan et al. https://creativecommons.org/licenses/by/4.0/ This content is distributed under the terms of the Creative Commons Attribution 4.0 International license.

**FIG 2:**
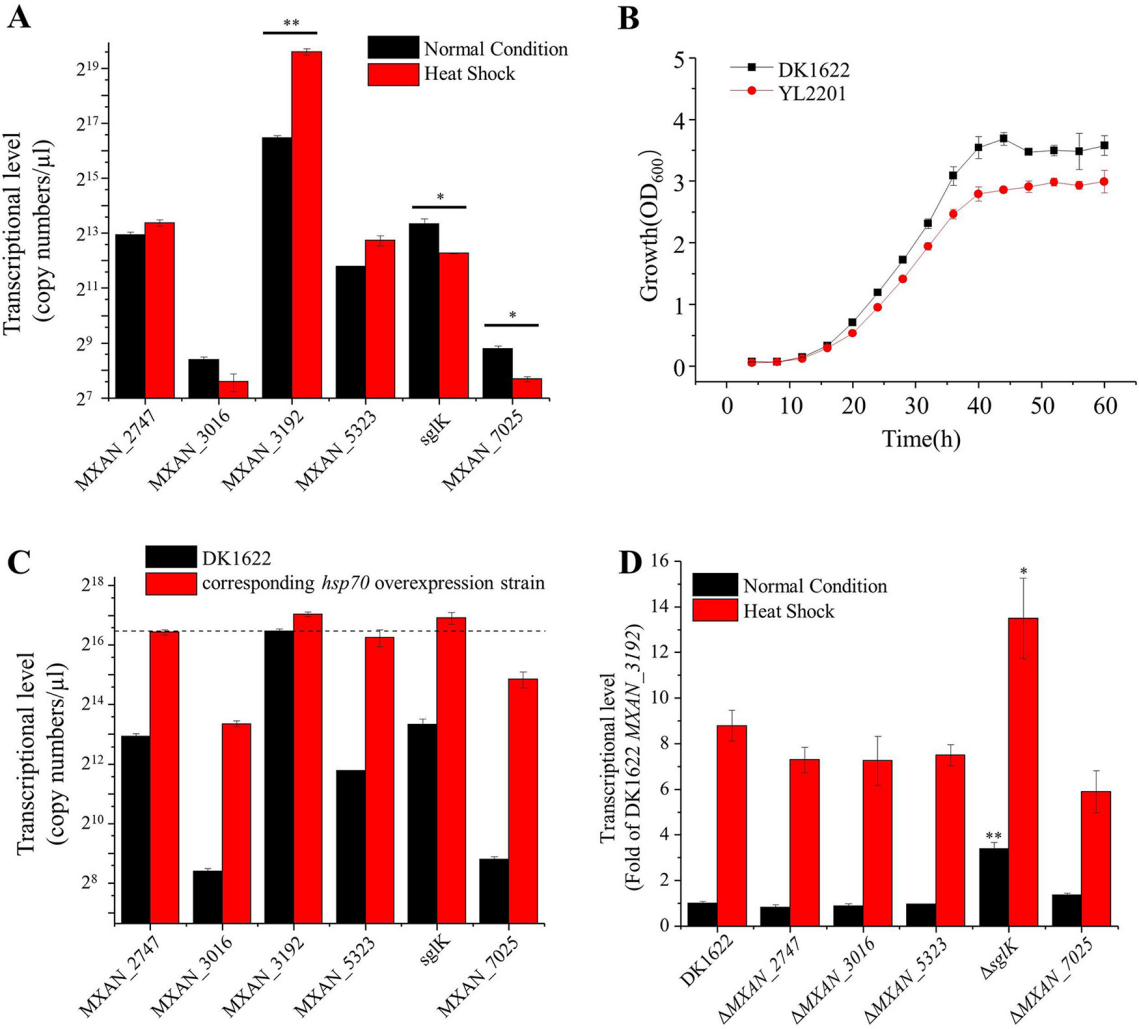
Quantitative PCR assays of *hsp70* transcription in M. xanthus DK1622 and the Hsp70 mutants. (A) Transcription of six *hsp70* genes in M. xanthus DK1622 under normal growth and heat shock conditions. (B) Comparison of growth curves of DK1622 and YL2201 mutants. (C) Transcriptional levels of different *hsp70* genes in DK1622 and the corresponding *hsp70* overexpression mutants. The dotted line represents the expression level of MXAN_3192 in DK1622. (D) MXAN_3192 expression in different *hsp70* deletion mutants. The expression level of MXAN_3192 in DK1622 under normal growth conditions was set to 1. Error bars represent standard deviations for three repeats. **, *P* < 0.01; *, *P* < 0.05.

To confirm the transcriptomics results, we further checked the transcriptional levels of the six *hsp70* genes using quantitative PCR under normal and heat shock conditions (Fig. 2A). Under normal temperature conditions, the transcription of MXAN_3192 was extremely high, far exceeding that of any of the other genes, which was consistent with our transcriptomics data. After 1 h of heat shock at 42°C, the transcription of MXAN_3192 increased approximately 8-fold, whereas the transcription of the other five *hsp70* genes showed no obvious increase; instead, some showed significantly decreased transcription after heat shock.

In E. coli, DnaK is nonessential for cell survival because of its functional redundancy with the trigger factor (TF), and only simultaneous deletion of *dnaK* and the TF gene is lethal (32). We attempted to knock out each of the six *hsp70* genes in DK1622 and successfully obtained deletion mutants of all the genes except MXAN_3192. To confirm the essentiality of MXAN_3192, we inserted a second copy of this gene at the Mx8 *attB* site, from which we successfully obtained the deletion mutant of the original MXAN_3192 gene (YL2201, which had an ectopically expressed MXAN_3192 gene but lacked the original MXAN_3192 gene). The results strongly suggested that MXAN_3192 is essential for cell survival. The YL2201 mutant exhibited a growth curve similar to that of wild-type M. xanthus DK1622 (Fig. 2B).

Our previous work determined that distinct phenotypes of the *groEL1* and *groEL2* mutants in response to heat shock were mainly due to the expression levels of the *groEL2* and *groEL1* genes in these two mutants (23). Therefore, we further inserted each of the five nonessential *hsp70* genes under the strong promoter P*_pilA_* (33) into the DK1622 genome at the *attB* site, producing five overexpression strains. Quantitative PCR assays indicated that the transcriptional levels of these genes were significantly increased in the corresponding overexpression strains; the expression levels of MXAN_2747, MXAN_5323, and *sglK* were similar to that of MXAN_3192, while those of MXAN_3016 and MXAN_7025 were low but still reached 30 to 50 times the original expression levels in DK1622 (Fig. 2C). We further tried to delete the MXAN_3192 gene in each of the overexpression strains, but all the attempts failed. The above results indicated that MXAN_3192 is unique among the Hsp70 homologues and probably plays an irreplaceable role in M. xanthus DK1622.

Interestingly, compared with DK1622, deletion of *sglK*, but not the other four *hsp70* genes, led to a significant increase in the transcription of MXAN_3192, and the level was approximately 3.4-fold or 1.5-fold higher than that in DK1622 under normal or heat shock conditions (Fig. 2D). We suggest that there might be a functional redundancy between MXAN_3192 and *sglK*, which led to MXAN_3192 being significantly upregulated after deletion of *sglK*.

### Inactivation of *hsp70* genes affects the social behaviors of M. xanthus.

**FIG 3:**
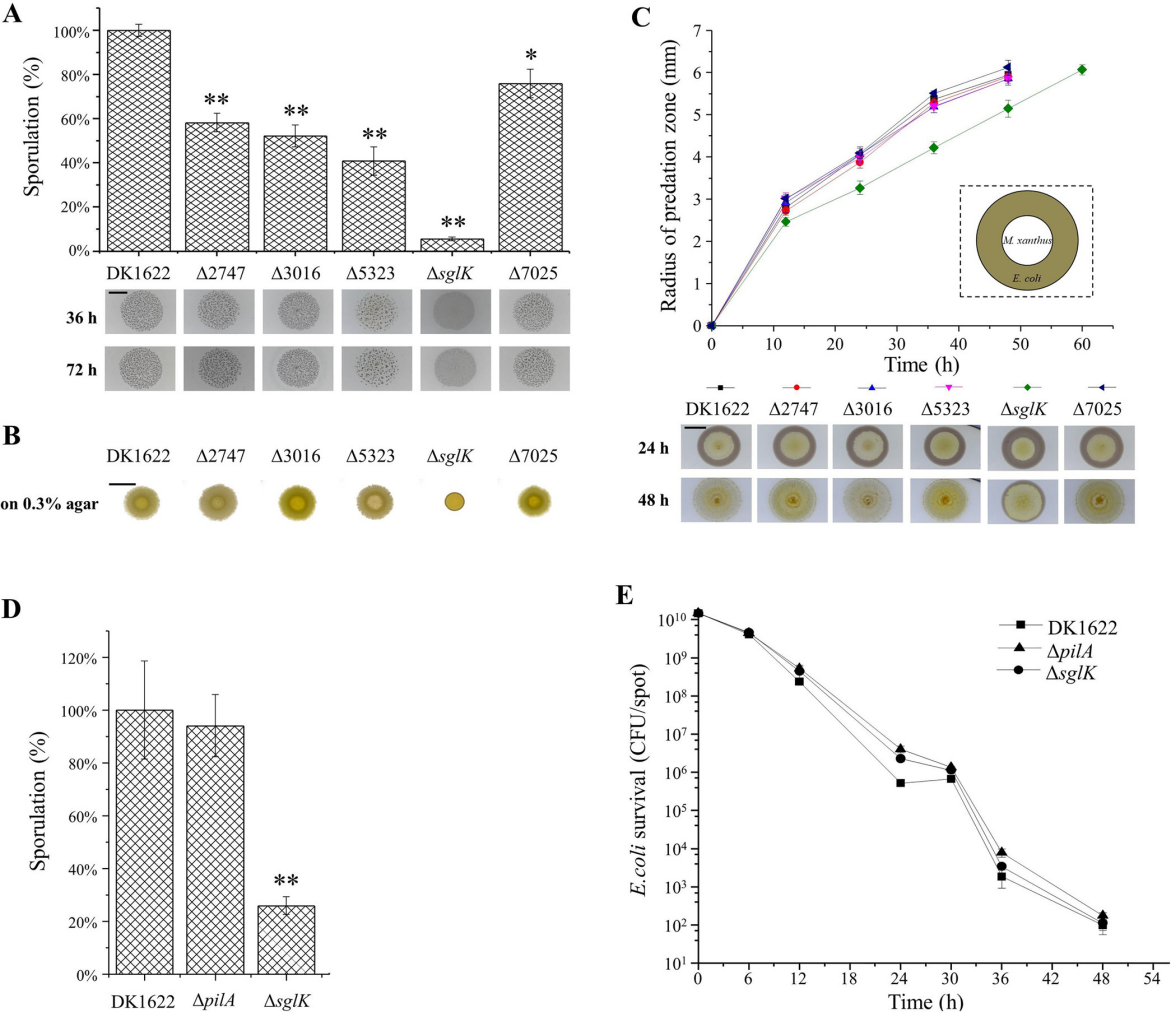
Comparison of the social characteristics of the *hsp70* deletion mutants with wild-type DK1622. (A) Starvation-induced sporulation ability (percentage of that in DK1622) and fruiting body development in *hsp70* deletion mutants. Cells were grown on TPM plates. Bar, 5 mm. **, *P* < 0.01; *, *P* < 0.05. (B) Motilities of different strains on 0.3% agar after 72 h of incubation. Bar, 10 mm. (C) Central predation feeding assays of different strains on an E. coli prey mat based on the radius of the predation zones. Bar, 5 mm. (D) Glycerol-induced sporulation of DK1622 and the Δ*sglK* and Δ*pilA* strains (percentage of that in DK1622). **, *P* < 0.01; *, *P* < 0.05. (E) Mixed predation feeding assay on E. coli cells based on the survival of E. coli cells.

Myxobacteria are characterized among prokaryotes by their social behavior and complex life cycle (20), e.g., myxobacteria cells glide on solid surfaces in a swarm, exhibit group predation and develop multicellular fruiting bodies that contain myxospores for long-term survival under starvation (34, 35). Previous studies showed that mutation of *sglK* by Tn5 insertion significantly affected the social motility and multicellular development of M. xanthus DK1622 cells (28, 29). We performed experimental assays of the social behaviors of the Hsp70 mutants. When incubated on starvation medium, the Δ*sglK* strain did not form viable fruiting bodies, which is consistent with previous results (28), while deletion of the other four *hsp70* genes had almost no effect on fruiting body formation. However, compared with DK1622, all five mutants showed different degrees of sporulation deficiency, among which the Δ*sglK* mutant had the lowest sporulation ability, which was approximately 5% of that of DK1622 (Fig. 3A).

M. xanthus cells have two motility systems: system A is required for the movement of single cells, and system S is mainly involved in the movement of cells in groups (36). We assayed the motilities of the Hsp70 mutants using DK1622, a Δ*aglZ* (A^−^ S^+^) mutant, and a Δ*pilA* (A^+^ S^−^) mutant as controls. Single cells of the Hsp70 mutants moved like wild-type cells on 1.5% agar plates (Fig. S3). However, deletion of *sglK* significantly decreased cellular S-motility on 0.3% agar (Fig. 3B), which is consistent with previous results (28). The other four mutants had swarming phenotypes similar to those of wild-type DK1622. Similarly, the central predation results demonstrated that the ΔMXAN_2747, ΔMXAN_3016, ΔMXAN_5323, and ΔMXAN_7025 mutants had nearly the same predation speed as the wild-type strain and reached the edge of the E. coli cell mat after approximately 36 h of incubation, whereas the inactivation of *sglK* significantly decreased the predation efficiency, so that the mutant reached the mat edge after 48 h of incubation (Fig. 3C).

10.1128/mSphere.00305-21.3 FIG S3 Comparison of A-motility of the *hsp70* mutants with DK1622 on 1.5% agar plate. (A) Morphologies of the edges of different colonies. A-motility is present as a single cell moves out a short distance from the edge of an expanding swarm. (B) Diameters of the swarming colonies. During a 3-day incubation, the swarming size of M. xanthus cells was measured and monitored every 12 h. The Δ*aglZ* (A^−^ S^+^) and Δ*pilA* (A^+^ S^−^) strains were included as controls. Δ and *att*:: represent gene deletion and overexpression mutants, respectively. Download FIG S3, TIF file, 2.2 MB. Copyright © 2021 Pan et al. 2021 Pan et al. https://creativecommons.org/licenses/by/4.0/ This content is distributed under the terms of the Creative Commons Attribution 4.0 International license.

It is known that M. xanthus cells need to move to accomplish development and predation, and core sporulation program occurs within fruiting bodies in the late stage of development (37, 38). To determine whether the effect of Δ*sglK* on sporulation and predation was caused by S-motility deficiency, we checked the sporulation and predation of the Δ*sglK* mutant when S-motility was bypassed, and a Δ*pilA* mutant defective in S-motility (39) was included as a control. For sporulation assays, we used glycerol to induce rapid M. xanthus spore production in liquid culture and compared the sporulation ability after 24 h of incubation. As shown in Fig. 3D, while the Δ*pilA* mutant exhibited sporulation ability similar to that of DK1622, the Δ*sglK* mutant was still significantly deficient in sporulation, indicating that SglK was required in the core sporulation program. For the predation assay, before spotting on plates, the M. xanthus cells were mixed sufficiently with the E. coli cells to allow thorough contact at the very beginning. After different incubation times, the survival of E. coli was recorded. Unlike sporulation, the Δ*sglK* mutant, as well as the Δ*pilA* mutant, had a predation efficiency similar to that of the DK1622 strain (Fig. 3E). The results demonstrated that deletion of *sglK* led to sporulation deficiency. However, the decreasing predation efficiency of the Δ*sglK* strain was due to the S-motility deficiency. Notably, although deletion of the *hsp70* genes might affect social behaviors, overexpression of these genes had almost no effect, except that the overexpression of *sglK* produced better S-motility than the wild-type strain (Fig. S3 and S4).

10.1128/mSphere.00305-21.4 FIG S4 Comparison of the social characteristics of the *hsp70* overexpression mutants with the wild-type DK1622. (A) Starvation-induced sporulation ability (percentage of that in DK1622) and fruiting body development in different *hsp70* deletion mutants. Cells were grown on TPM plates. Bar, 5 mm. **, *P* < 0.01; *, *P* < 0.05. (B) Motilities of different strains on 0.3% agar after 72 h of incubation. Bar, 10 mm. (C) Central predation feeding assays of different strains on an E. coli prey mat based on the radius of the predation zones. Bar, 5 mm. Download FIG S4, TIF file, 2.3 MB. Copyright © 2021 Pan et al. 2021 Pan et al. https://creativecommons.org/licenses/by/4.0/ This content is distributed under the terms of the Creative Commons Attribution 4.0 International license.

### Hsp70s are involved in the oxidation resistance of M. xanthus DK1622.

**FIG 4:**
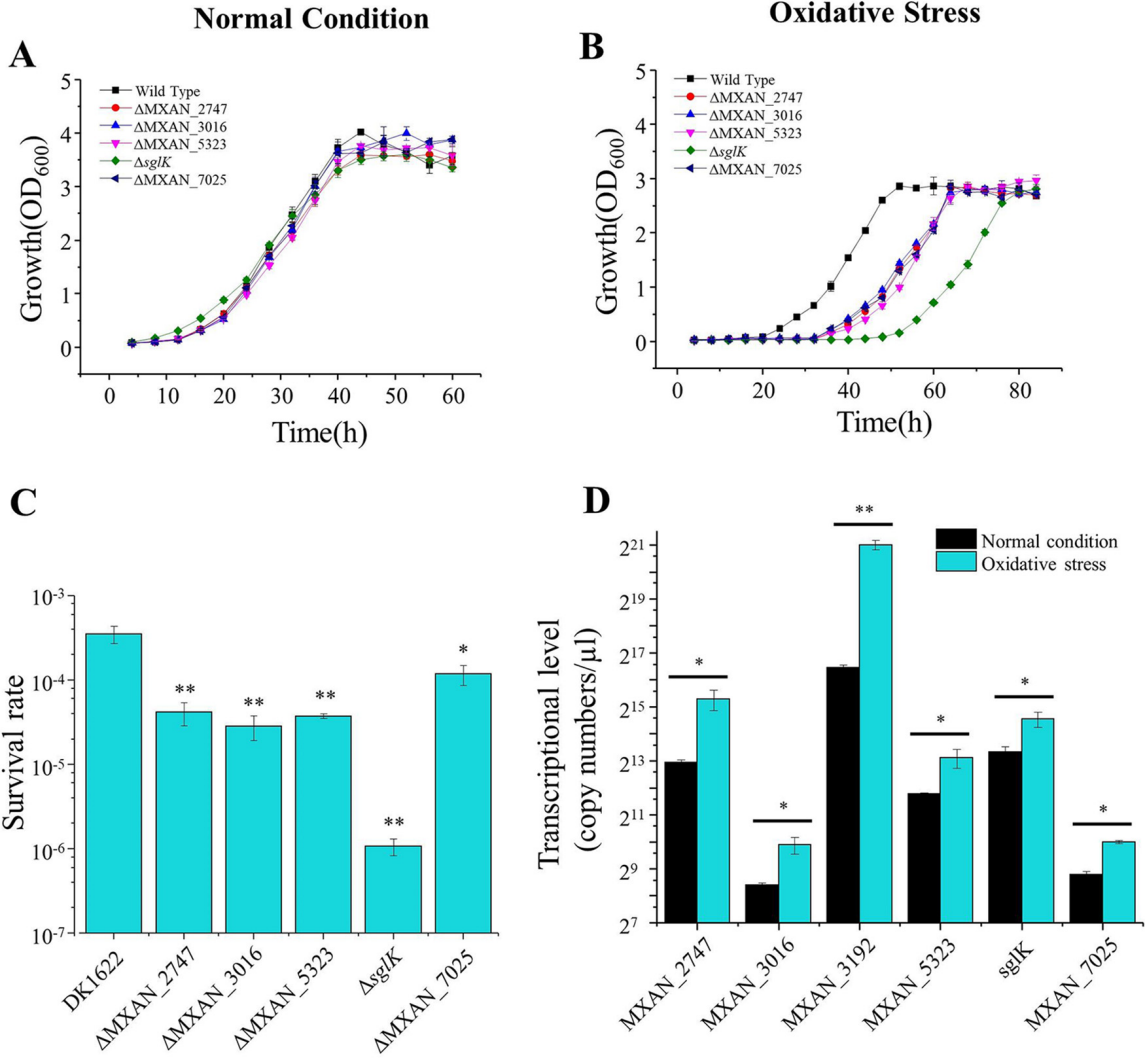
Growth curves of *hsp70* deletion mutants under normal growth (A) and oxidative stress (B) conditions. (C) Survival rates of different mutants after H_2_O_2_ treatment. (D) qPCR analysis of *hsp70* transcription in M. xanthus DK1622 under normal growth and oxidative stress conditions. Oxidative stress was induced by treating M. xanthus cells with 1.5 mM hydrogen peroxide (H_2_O_2_) for 30 min before inoculation. Error bars show standard deviations for three repeats. **, *P* < 0.01; *, *P* < 0.05.

DnaK expression is normally inducible by various environmental stresses, such as heat shock, oxidation, and osmotic damage, thus preventing proteins from aggregating or refolding proteins damaged by stress (7, 17). Absence of the corresponding genes usually causes delayed growth under stress. We assayed the growth of five Hsp70 deletion mutants under environmental stress, that is, high temperature, low temperature, high osmotic pressure, or oxidative stress. The different deletion mutants showed almost the same growth curve as wild-type DK1622 under the normal growth conditions (Fig. 4A). However, when treated with 1.5 mM H_2_O_2_ before inoculation, compared with wild-type DK1622, all five Hsp70 deletion mutants showed delayed growth but reached almost the same final cell density after 72 h of incubation (Fig. 4B). The growth lag phase is an important period during which bacterial cells address environmental changes and protein misfolding accumulated during cell arrest (40). Inactivation of Hsp70s caused cells to spend more time repairing oxidative damage. Among the five Hsp70 mutants, the Δ*sglK* strain had the longest lag phase. We further assayed the survival rates of these mutants in response to oxidative stress and found that the survival rates of the mutants were all significantly lower than that of DK1622. Similarly, the survival rate of the Δ*sglK* mutant was also significantly lower than that of the other mutants (Fig. 4C). These results suggested that all five nonessential Hsp70s participated in the oxidation resistance process, and SglK played a more important role than the others.

We further measured the growth of the *hsp70* overexpression mutants under normal and oxidative stress conditions. The growth curves of these overexpression mutants were almost identical to that of the wild-type strain (Fig. S5A and B). The results suggested that the original expression levels of these genes were probably sufficient for the recovery of oxidative damage. We assayed the transcriptional levels of the *hsp70* genes in wild-type DK1622 in response to oxidative damage by using quantitative PCR. Treatment with H_2_O_2_ for 30 min resulted in dramatically increased expression of MXAN_3192 in DK1622 cells; the expression level was approximately 25-fold that under normal conditions (*P <* 0.01) (Fig. 4D). The expression of the other five *hsp70* genes was also significantly increased under oxidative stress (*P <* 0.05) but less obviously than that of MXAN_3192. Combined with the growth curves and survival rates of the deletion mutants, the above results suggested that each of these Hsp70s is involved in the oxidation resistance of M. xanthus DK1622. However, single deletions of the five *hsp70* genes had no obvious impact on the growth under high temperature, low temperature, and high osmotic pressure (Fig. S5C to E).

10.1128/mSphere.00305-21.5 FIG S5 Assays of the growth abilities of *hsp70* mutants under different conditions. (A) Overexpression mutants with the normal growth temperature. (B) Overexpression mutants after heat shock. (C) Deletion mutants under high osmotic pressure (1.5% mannitol). (D) Deletion mutants at high temperature (35.5°C). (E) Deletion mutants at low temperature (26°C). Error bars show standard deviations for three repeats. Download FIG S5, TIF file, 1.2 MB. Copyright © 2021 Pan et al. 2021 Pan et al. https://creativecommons.org/licenses/by/4.0/ This content is distributed under the terms of the Creative Commons Attribution 4.0 International license.

### MXAN_3192 or MXAN_6671 restores the growth deficiency of E. coli caused by *dnaK* deletion.

**FIG 5:**
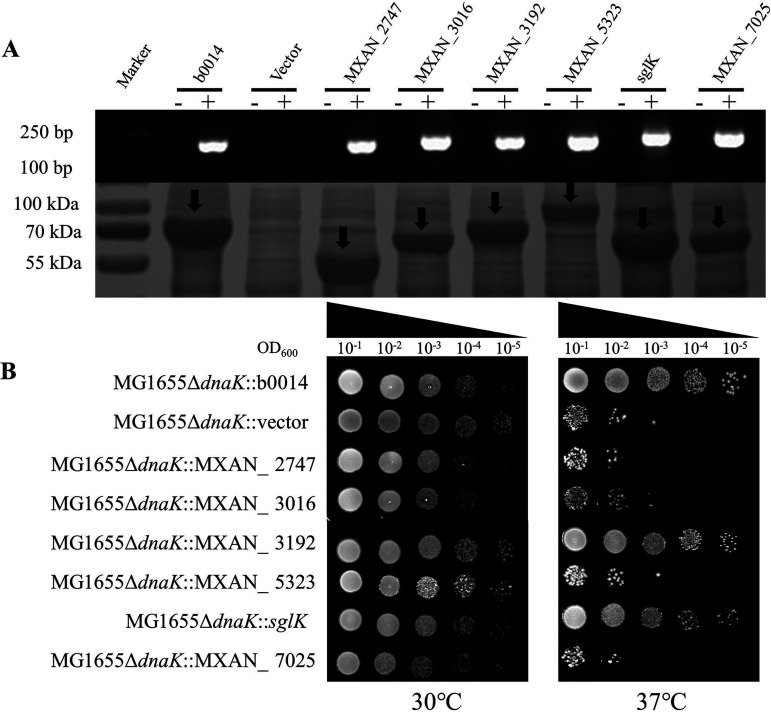
Growth test of the E. coli *dnaK* deletion mutants complemented with different *Myxococcus hsp70* genes. (A) DNA transcription from real-time PCR with the corresponding primers (top; −, RNA samples without reverse transcription; +, cDNA samples) and protein expression in an SDS-polyacrylamide gel (bottom) of complemented *hsp70* genes in different E. coli cells. (B) Growth test of the E. coli *dnaK* deletion mutants complemented with different *hsp70* genes from M. xanthus at 30°C or 37°C.

Although *dnaK* is nonessential for E. coli, deletion of it leads to an obvious cell growth deficiency at 37°C, which can be overcome by compensating with an active *dnaK* gene (41, 42). We compensated for the deficiency with six *Myxococcus hsp70* genes in an E. coli *dnaK* deletion mutant and assayed the growth abilities of the compensation strains. Real-time PCR (RT-PCR) and SDS-PAGE showed that the *Myxococcus hsp70* genes were transcribed and translated normally in the E. coli strain as the compensated E. coli *dnaK* gene (the gene locus tag was *b0014*, and the product was EcDnaK) (Fig. 5A). When incubated at 30°C, the six recombinant strains displayed a growth ability similar to that of the positive and negative controls. However, at 37°C, the MXAN_3192 and *sglK* recombinant strains showed a growth ability similar to that of the *b0014* compensation strain and much better than that of the strain harboring an empty vector, whereas the growth of the other four mutants was much weaker than that of the *b0014* compensation strain (Fig. 5B). The results indicated that MXAN_3192 or SglK is able to compensate for the role of EcDnaK in E. coli growth at 37°C.

## DISCUSSION

Hsp70 has multiple copies in eukaryotic cells, and the multiple Hsp70s are characterized by differences in expression in terms of tissue-specific abundance and subcellular localization, resulting in functional diversification of different Hsp70 isoforms (12, 26). In this study, we found that many prokaryotes also have more than one gene encoding Hsp70 proteins, such as myxobacteria. The Hsp70 family proteins in bacteria can be divided into different types according to the conserved domains of cl35085 (PRK00290) and cd10170. Many previous studies have focused on heat shock-inducible DnaK proteins (containing the PRK00290 domain), while the functions of other Hsp70s in bacteria have been less well explored. In M. xanthus DK1622, there are two genes (MXAN_3192 and *sglK*) encoding proteins containing the PRK00290 conserved domain and many genes encoding proteins with the cl35085 (but not PRK00290) or cd10170 domain. We found that Hsp70s with a cl35085 or cd10170 domain (MXAN_2747, MXAN_3016, MXAN_5323, and MXAN_7025) exhibited some similar functions in the physiological processes of M. xanthus DK1622, for example, in sporulation and oxidation resistance. The results suggested that these multiple noncanonical Hsp70s have similar functions in these physiological processes, similar to the SSA family proteins in S. cerevisiae (43); they probably evolved as helpers with some unknown specific functions in the complex cellular life cycle. For example, in a transcriptomic study of the 96-h developmental program, MXAN_2747, MXAN_5323, and *sglK* were significantly upregulated in the developmental process and exhibited peak expression in the aggregation or sporulation phase; however, MXAN_3192, MXAN_3016, and MXAN_7025 were not available in the transcriptomic analysis (44).

Probably because of its functional redundancy with TF or GroEL in the folding of *de novo*-synthesized proteins or denatured proteins under stress conditions, DnaK is nonessential in some bacteria, such as Acinetobacter baumannii and E. coli (5, 32). In some other cases, DnaK also plays an essential role in cell growth, such as in Caulobacter crescentus, Mycobacterium smegmatis, and *Synechococcus* sp. (14, 40, 41), but probably for different reasons. For example, in C. crescentus, DnaK was found to be related to the degradation of DnaA by the Lon protease, which results in cell cycle arrest (45), while in M. smegmatis, DnaK was found to be required for solubility of the essential lipid synthase FASI (46). In M. xanthus DK1622, proteins with the PRK00290 conserved domain play a central role in complex cellular functions. MXAN_3192 not only had a high transcriptional level and was inducible by heat shock but was also the only undeletable *hsp70* gene in M. xanthus DK1622. We determined that upregulating other Hsp70s to a similar transcriptional level of MXAN_3192 could not replace the gene for cell viability.

The second PRK00290 protein, Sglk, also seems to be more important than the other Hsp70 proteins. For example, deletion of MXAN_6671 resulted in the lowest sporulation ability, the lowest survival rates, and the longest lag phase after H_2_O_2_ treatment compared with the other four assayed *hsp70* genes. Unlike MXAN_3192, *sglK* does not exhibit extremely high expression in M. xanthus DK1622. Moreover, heat shock led to significantly decreased expression of *sglK*, in contrast to the increased expression of the canonical DnaK (4). Notably, probably due to their phylogenetic and functional similarity to EcDnaK, the PRK00290 proteins MXAN_3192 and SglK can recover the growth deficiency of the E. coli *dnaK* deletion mutant. We speculate that although the two PRK00290 proteins both retain some fundamental functions, they are functionally different; SglK evolved distantly from the typical DnaK, and MXAN_3192 retained more conserved classical DnaK functions. Thus, it will be interesting to further explore the divergence mechanisms between MXAN_3192 and SglK.

## MATERIALS AND METHODS

### Cultures, plasmids, and growth conditions.

The strains, plasmids, and primers used in this study are listed in Tables S2 and S3. The E. coli strains were routinely grown on Luria-Bertani (LB) agar or in LB liquid broth at 37°C. The M. xanthus strains were cultivated in CTT medium (47) at 30°C. When required, 40 μg/ml kanamycin, 100 μg/ml ampicillin, or 10 μg/ml tetracycline (final concentrations; Solarbio, China) was added to the solid or liquid medium.

10.1128/mSphere.00305-21.9 TABLE S2 Bacterial strains and plasmids used in this study. Download Table S2, DOCX file, 0.02 MB. Copyright © 2021 Pan et al. 2021 Pan et al. https://creativecommons.org/licenses/by/4.0/ This content is distributed under the terms of the Creative Commons Attribution 4.0 International license.

10.1128/mSphere.00305-21.10 TABLE S3 Primers used in this study. Download Table S3, DOCX file, 0.02 MB. Copyright © 2021 Pan et al. 2021 Pan et al. https://creativecommons.org/licenses/by/4.0/ This content is distributed under the terms of the Creative Commons Attribution 4.0 International license.

### Bioinformatics analysis of the occurrence of *hsp70* genes in prokaryotic genomes.

Conserved domain information was obtained from the CDD protein families (cd10170, HSP70_NBD; cl35085, DnaK superfamily; and PRK00290, DnaK) (11). Based on the conserved domains of cl35085 (PRK00290) and cd10170, we retrieved all Hsp70 protein sequences from 5,551 representative prokaryotic genomes (30, 48).

The six *hsp70* genes selected from the M. xanthus DK1622 genome for experiments are MXAN_2747 (MXAN_RS13310), MXAN_3016 (MXAN_RS14605), MXAN_3192 (MXAN_RS15460), MXAN_5323 (MXAN_RS25830), MXAN_6671 (MXAN_RS32305), and MXAN_7025 (MXAN_RS34005), and the corresponding NCBI accession numbers for the protein sequences are WP_011552814.1, WP_011553072.1, WP_011553239.1, WP_011555288.1, WP_011556596.1, and WP_011556944.1. The typical DnaK protein sequences from E. coli, B. subtilis, and L. lactis were obtained from the NCBI protein database.

We used MEGA and the online CVTree3 program (49) to construct phylogenetic trees based on protein and whole-genome sequences. The phylogenetic trees were annotated by iTOL online (50). Multiple sequence alignment was generated by using the online MAFFT program and analyzed with ESPript (51).

### Deletion of *hsp70* genes.

Deletion of *hsp70* genes in M. xanthus was performed using positive-negative KG cassettes (52). Genomic DNA from DK1622 served as a template for the PCR amplification of the upstream and downstream homologous arms utilizing Phanta Super-Fidelity DNA polymerase (Vazyme, China). The arms were fused to create homologous sequences and cloned into pBJ113 to form deletion plasmids, which were transferred via electroporation into M. xanthus DK1622 cells as previously described (39). Individual kanamycin-resistant colonies were selected and inoculated onto CTT agar plates supplemented with 1.5% galactose (Sigma, USA) for a second round of screening. The deletion mutants were identified based on their kanamycin sensitivity and galactose resistance phenotypes, as well as by PCR and sequencing verification. Notably, after two rounds of selection, the kanamycin resistance gene was removed, and the mutants did not harbor kanamycin resistance.

Deletion of the *dnaK* gene in E. coli MG1655 was conducted according to a previously published method (53) with slight modification; i.e., we did not eliminate the resistance cassette from the transformants, and the deletion mutants thus still exhibited kanamycin resistance.

### Overexpression of genes in M. xanthus.

The overexpression vector was constructed by using the suicide plasmid pSWU30 and the promoter of the *pilA* gene of DK1622. The promoter of the *pilA* gene (630 bp) was amplified using Phanta Super-Fidelity DNA polymerase (Vazyme, China) and constructed by fusion with the *hsp70* gene to be overexpressed. The fragment was further fused with the homologous arms with XbaI/EcoRI sites and then inserted into pSWU30 to form an overexpression plasmid. After electroporation, the plasmid was integrated at the Mx8 *attB* site in the genome of M. xanthus DK1622. The mutants were selected with 10 μg/ml tetracycline and verified by PCR and sequencing.

### Quantitative real-time PCR analysis.

Real-time quantitative PCR (RT-qPCR) was performed as previously described with minor modifications (23). Briefly, M. xanthus DK1622 cells and mutants were collected from 24-h cultures and inoculated into fresh CTT medium at a final concentration of 1 × 10^7^ cells/ml. To initiate a heat shock reaction, the cells were cultured at 42°C for an additional 1 h, and then RNA was extracted using a bacterial RNA extraction kit (Thermo Fisher, USA). After removal of genomic DNA, the purified RNA extracts were immediately reverse transcribed to cDNA, which was stored at −80°C. RT-qPCR was performed with a total reaction volume of 20 μl containing 2 μl of primers at 100 nM, 10 μl of SYBR green PCR master mix (TaKaRa, Japan), 7 μl of RNase-free water, and 1 μl of a 5-fold-diluted cDNA template. PCR was conducted as follows: 3 min at 95°C, followed by 40 cycles of 30 s at 95°C, 30 s at 55°C, and 15 s at 72°C. The target genes were PCR amplified, purified, and then serially diluted to at least nine concentrations to calculate their copy numbers. After RT-qPCR, the standard curve was plotted using the copy numbers and cycle threshold (*C_T_*) values. The *gapA* gene (a glyceraldehyde-3-phosphate dehydrogenase-encoding gene, MXAN_2815) was used as the reference to normalize the amount of cDNA. Gene expression was calculated using the standard curve. The primers used for RT-qPCR are listed in Table S2.

### Predation assays.

The central predation assay was performed according to the method used in a previous study (54) with slight modification. Briefly, E. coli and M. xanthus cultures were harvested at the mid-logarithmic phase and washed three times with TPM buffer (10 mM Tris-HCl, 8 mM MgSO_4_, 1 mM K_2_HPO_4_-KH_2_PO_4_ [pH 7.6]) (47). The E. coli and M. xanthus cells were concentrated to a final density of 1 × 10^11^ cells/ml and 5 × 10^9^ cells/ml, respectively. Then, 35 μl of E. coli was pipetted onto a plate to form a cell mat, and 5 μl of M. xanthus was added to the center of the mat. The plates were incubated at 30°C for 60 h, and the distance from the edge of the M. xanthus colony to the edge of the E. coli mat was recorded every 12 h.

In the mixed-predation assay, the E. coli and M. xanthus cells were separately harvested and washed as described above. Then, the M. xanthus cells were mixed with E. coli at a ratio of 1:1,000, and 37 μl of the mixture was pipetted onto TPM starvation medium. The plates were incubated at 30°C for 48 h, and the cell mixture was collected, vortexed thoroughly, serially diluted, and spread onto LB plates. The predation ability was measured by monitoring the viability of E. coli cells.

### Developmental assays.

M. xanthus cells were harvested at mid-logarithmic phase and resuspended in TPM buffer to a final concentration of 5 × 10^9^ cells/ml. Aliquots (8 μl) were dropped onto TPM agar. The cultures were incubated at 30°C and detected every 12 h under a dissection microscope. Sporulation was measured on 5-day TPM cultures as described previously (22). Fruiting bodies were harvested in 1 ml of TPM buffer and incubated at 50°C for 2 h to kill vegetative cells. After sonication, the spore suspensions were serially diluted and plated on CTT agar. After 5 days, the sporulation rate was calculated as the number of colonies.

The rapid sporulation assay was performed as described elsewhere (55). Briefly, the cells in exponential phase were harvested and washed with cold CTT. Then, the cells were inoculated into liquid medium containing 1% Casitone, 8 mM MgSO_4_, and 0.5 M glycerol and incubated at 30°C. After 24 h, the glycerol-induced spores were harvested, incubated at 50°C for 2 h, serially diluted, and plated on CTT agar. The CFU were counted after 5 days of incubation.

### Swarm assays.

The swarm assays were performed according to the method used in a previous study (36). Briefly, M. xanthus cultures were harvested at mid-logarithmic phase, washed three times with TPM buffer (pH 7.6), and adjusted to a final cell concentration of 5 × 10^9^ cells/ml. Aliquots (2 μl) were dropped onto 1.5% CTT agar and 0.3% CTT agar. After 72 h of incubation, the swarming size of M. xanthus cells was monitored.

### Growth and resistance analysis.

M. xanthus strains were grown in CTT medium with shaking at 200 rpm at 30°C to the mid-logarithmic phase (optical density at 600 nm [OD_600_] of ∼1) as seed liquid. Then, the cells were inoculated at an OD_600_ of 0.04 and grown in CTT medium for 60 h with shaking at 200 rpm. The OD_600_ value was read every 4 h.

To assay the growth under various environmental stresses, we performed experiments with corresponding modifications. For the high- or low-temperature stresses, the growth assay was determined by growing cells in liquid medium at 35.5°C or 26°C; for the high osmotic pressure, a final concentration of 1.5% mannitol was added to CTT medium, and for the oxidative damage assay, cells were treated with 1.5 mM hydrogen peroxide (H_2_O_2_) for 30 min before inoculation. The culture time was accordingly extended to 84 h.

### Oxidative damage assays.

M. xanthus cells were grown to the mid-logarithmic phase as described above. The cells were harvested, serially diluted, and plated on CTT agar before or after hydrogen H_2_O_2_ treatment. After 5 days of incubation, the survival rate was estimated as the number of CFU with oxidative stress over the number of CFU without oxidative stress. Assays were performed with three biological replicates.

### Growth test of E. coli.

Each of the six *hsp70* genes from M. xanthus was introduced into the expression plasmid pTrac99a using the ClonExpress II One Step cloning kit (Vazyme, China). The plasmids were transformed into the E. coli *dnaK* deletion mutant. The recombinant mutants were cultured overnight at 30°C and suspended in LB medium at an OD_600_ of 1. After gradient dilution, aliquots (3 μl) were dropped onto LB agar containing 40 μg/ml kanamycin, 100 μg/ml ampicillin, and 1 mM isopropyl-β-d-thiogalactopyranoside (IPTG). To test the expression level of Hsp70s, overnight cultures of fresh transformants were inoculated into the aforementioned LB medium without agar. After shaking at 37°C for 3 h, E. coli cells were harvested. Total RNA was extracted from half the volume of the cells and reverse transcribed to cDNA for RT-PCR as described above. The other cells were sonicated on ice and cleared by high-speed centrifugation (12,000 × *g*, 30 min) to obtain the total proteins in the supernatant. After quantification, equal amounts of proteins from different recombination mutants were loaded onto SDS-PAGE gels.

### Statistical analysis.

The difference significance was analyzed statistically based on paired *t* test (two-tailed).
