# Supplementary figures and images for: Bioinformatic and Functional Characterization of Hsp70s in Myxococcus xanthus
Source: mSphere. 2021 May 19;6(3):e00305-21. doi: 10.1128/mSphere.00305-21 (PMC8265645; doi:10.1128/mSphere.00305-21)

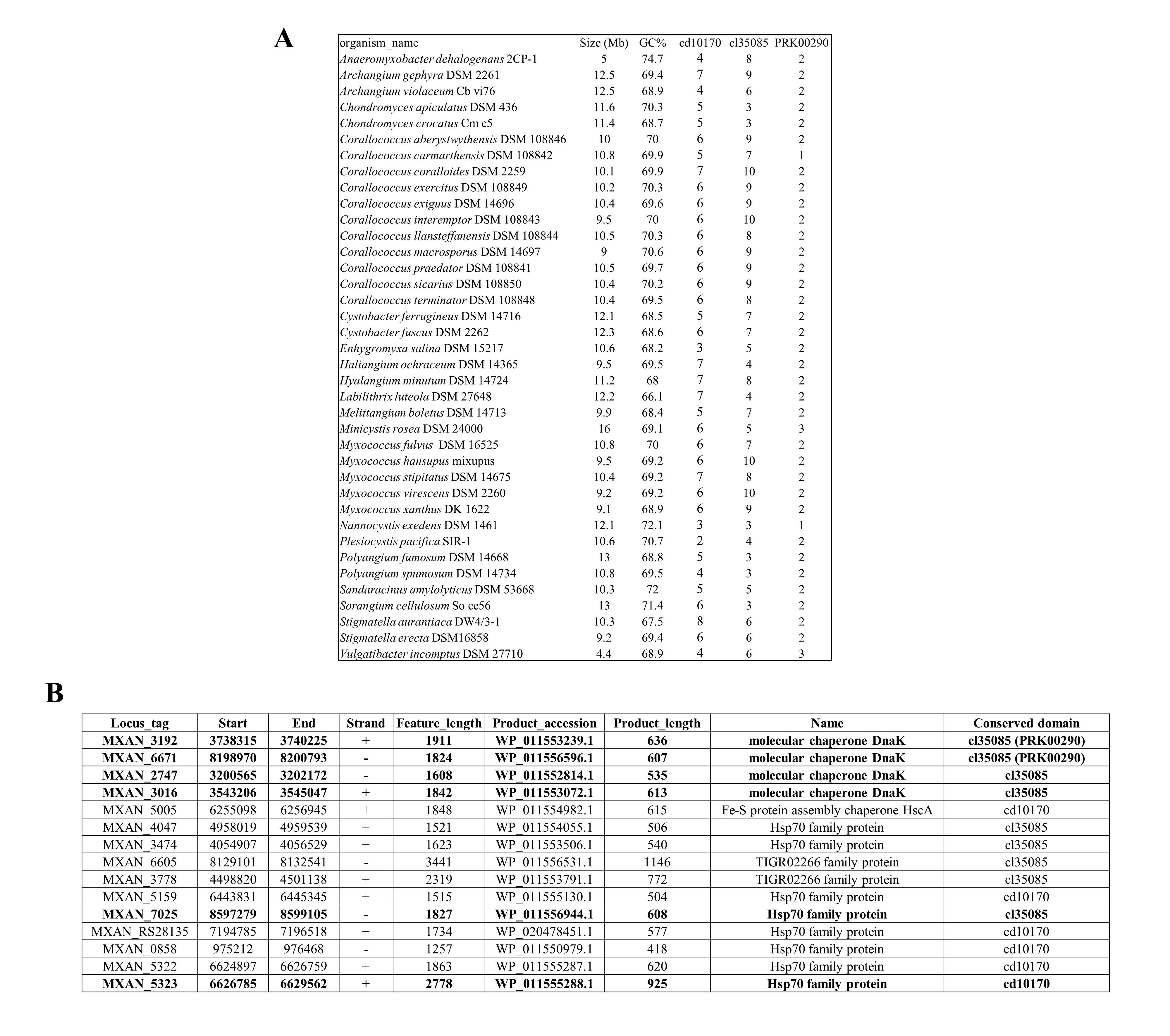

Supplement: TABLE S1 [file msphere.00305-21-st001.tif]

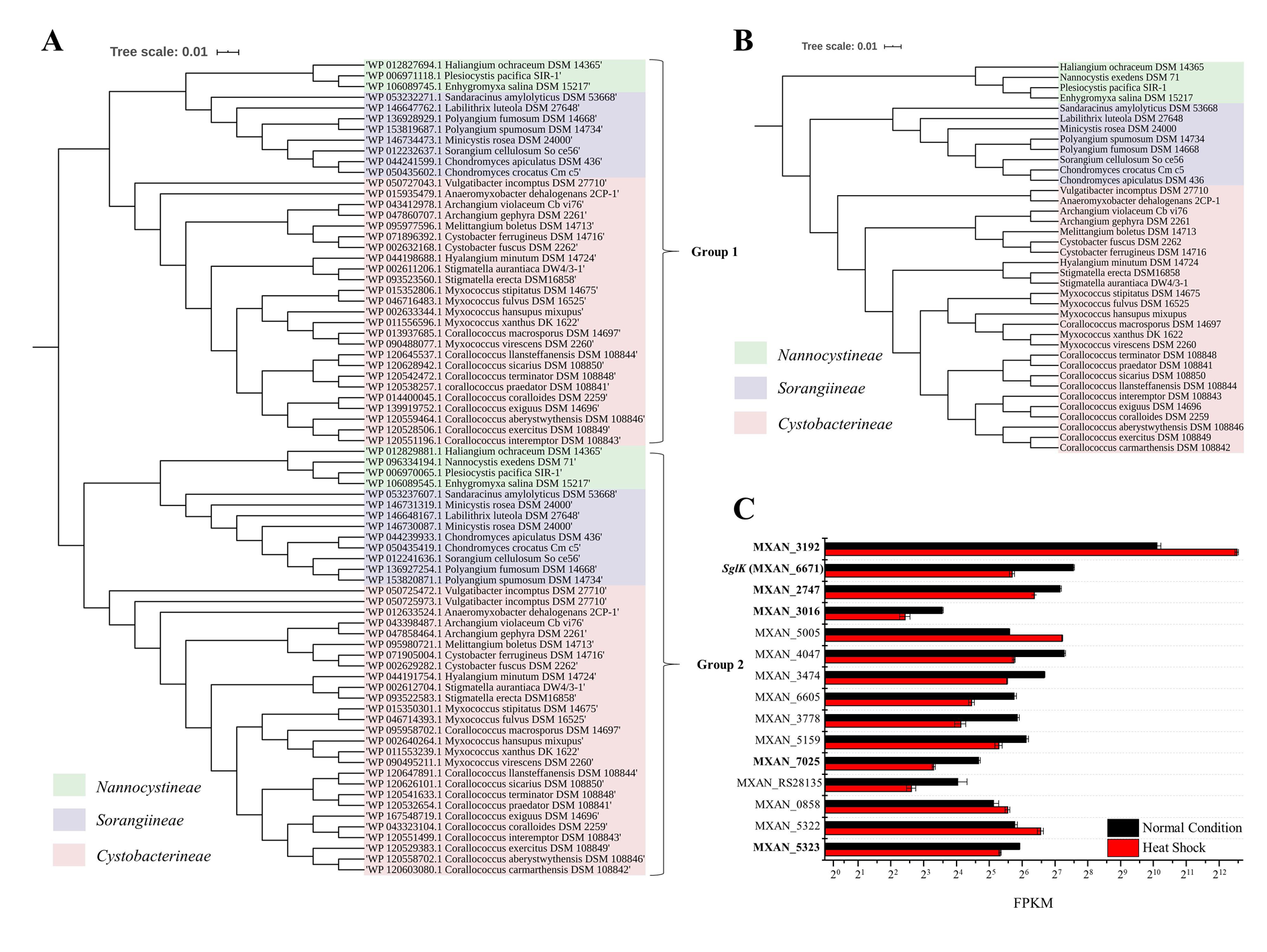

Supplement: FIG S1 [file msphere.00305-21-sf001.tif]

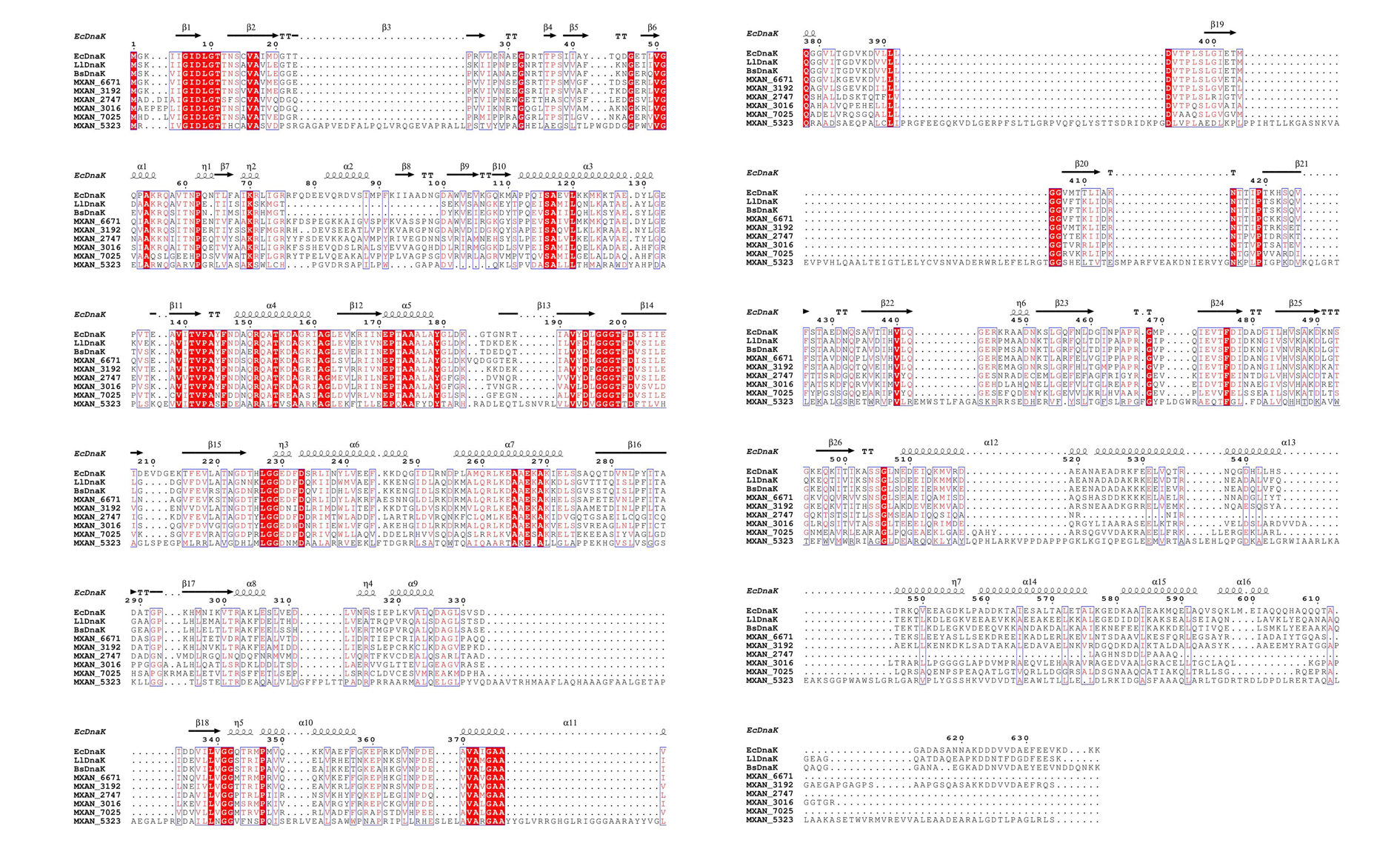

Supplement: FIG S2 [file msphere.00305-21-sf002.tif]

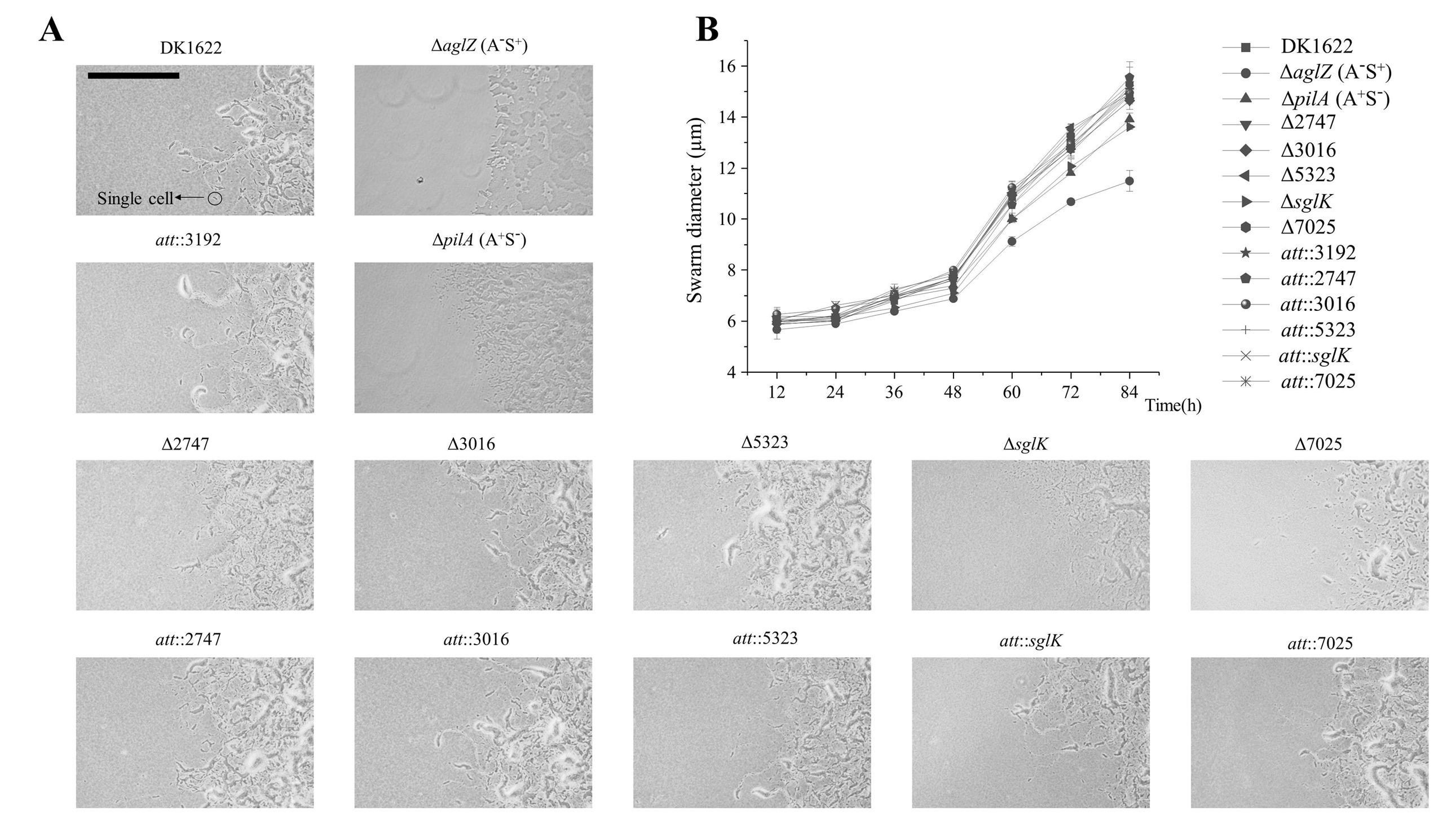

Supplement: FIG S3 [file msphere.00305-21-sf003.tif]

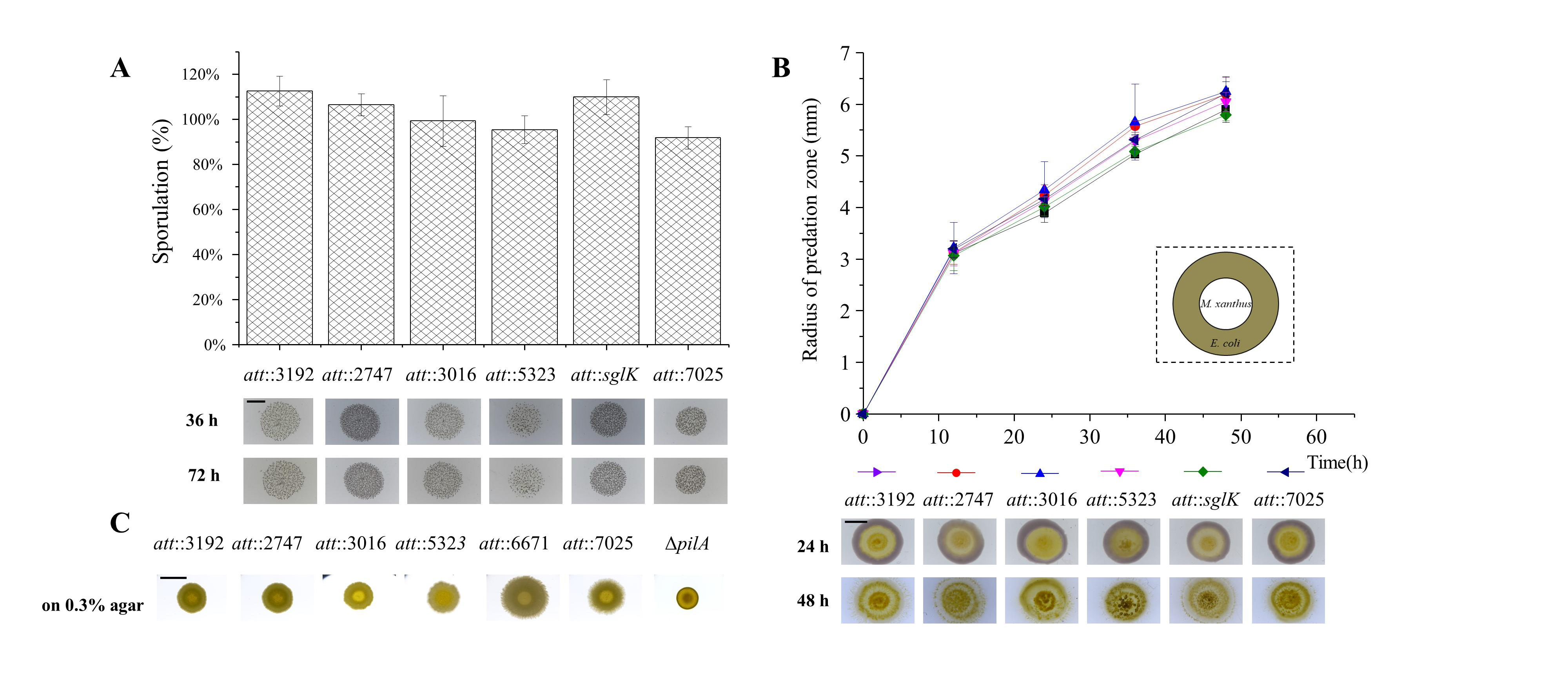

Supplement: FIG S4 [file msphere.00305-21-sf004.tif]

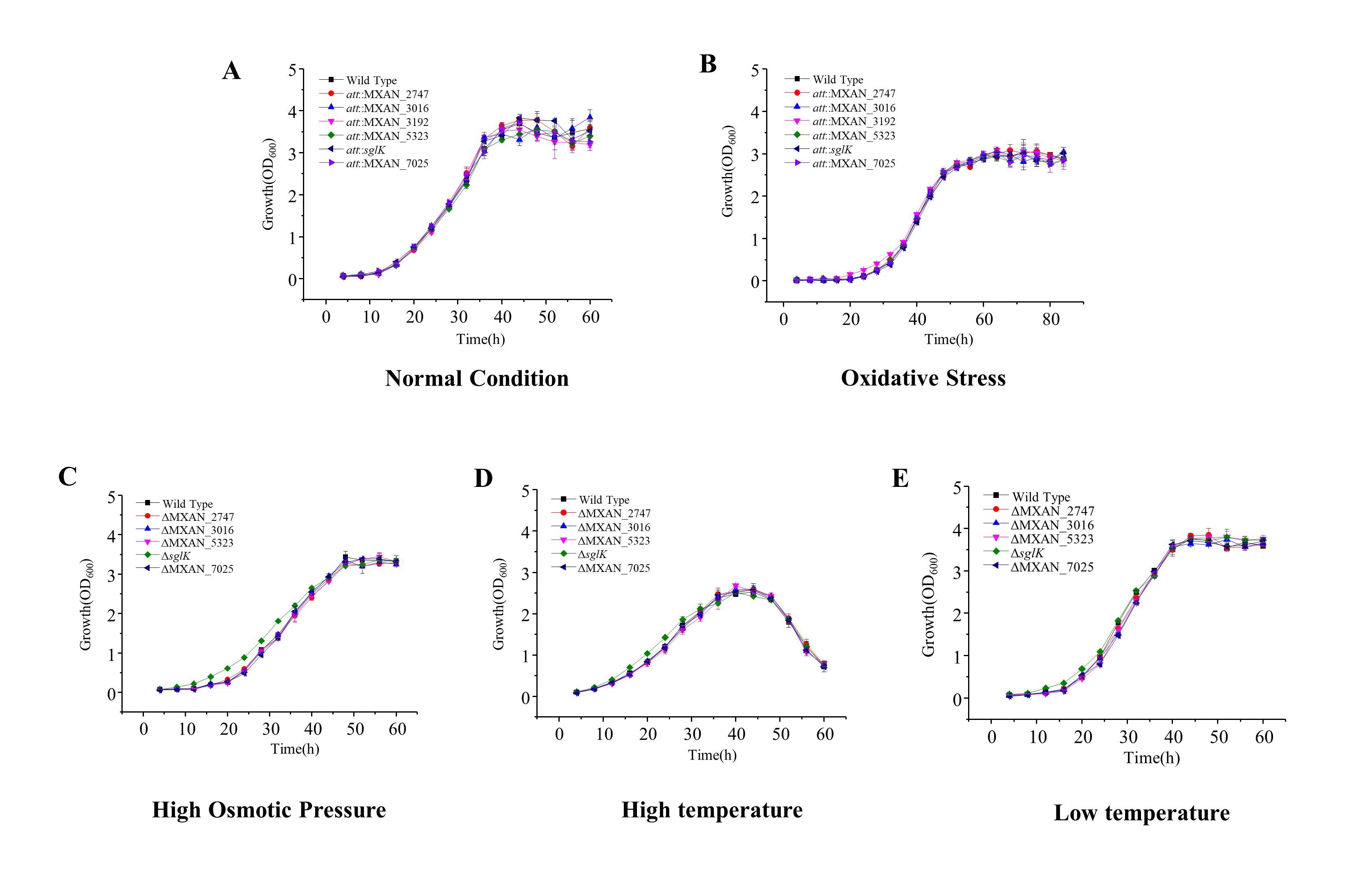

Supplement: FIG S5 [file msphere.00305-21-sf005.tif]
