# Supplementary material for: Bioinformatic and Functional Characterization of Hsp70s in Myxococcus xanthus
Source: mSphere. 2021 May 19;6(3):e00305-21. doi: 10.1128/mSphere.00305-21 (PMC8265645; doi:10.1128/mSphere.00305-21)
Supplement: TABLE S2 [file msphere.00305-21-st002.docx]

**Table S2.** Bacterial strains and plasmids used in this study.

| Strain and plasmid | Genotype or description | Source or reference |
| --- | --- | --- |
| Strains | | |
| M. xanthus | | |
| DK1622 | Wild-type strain | D.Kaiser University of Standford |
| Δ*pilA* | DK1622 Δ*MXAN_5783* (DK10410) | D.Kaiser University of Standford |
| Δ*aglZ* | DK1622 Δ*MXAN_2991* (MxH2265) | P. Hartzell  University of Idaho |
| Δ*MXAN_2747* | DK1622 Δ*MXAN_2747* | This study |
| Δ*MXAN_3016* | DK1622 Δ*MXAN_3016* | This study |
| Δ*MXAN_5323* | DK1622 Δ*MXAN_5323* | This study |
| Δ*sglK* | DK1622 Δ*MXAN_6671* | This study |
| Δ*MXAN_7025* | DK1622 Δ*MXAN_7025* | This study |
| *att*:: *MXAN_2747* | DK1622::pSWU30- *MXAN_2747* (*MXAN_2747* integrated at attB site with pilA promoter) | This study |
| *att*:: *MXAN_3016* | DK1622::pSWU30-*MXAN_3016* (*MXAN_3016* integrated at attB site with pilA promoter*)* | This study |
| *att*:: *MXAN_3192* | DK1622::pSWU30 *MXAN_3192* (*MXAN_3192* integrated at attB site with pilA promoter*)* | This study |
| *att*:: *MXAN_5323* | DK1622::pSWU30-*MXAN_5323* (*MXAN_5323* integrated at attB site with pilA promoter*)* | This study |
| *att*:: *sglK* | DK1622::pSWU30-*MXAN_6671* (*MXAN_6671* integrated at attB site with pilA promoter*)* | This study |
| *att*:: *MXAN_7025* | DK1622::pSWU30-*MXAN_7025* (*MXAN_7025* integrated at attB site with pilA promoter*)* | This study |
| YL2201 | DK1622::pSWU30-*MXAN_3192* Δ*MXAN_3192* | This study |
| **E. coli** | | |
| MG1655 | F- λ- ilvG- rfb-50 rph-1 | Stratagene |
| MG1655Δ*dnaK*:: *MXAN_2747* | pTrc99a- *MXAN_2747* plasmid transformed into MG1655Δ*dnaK* | This study |
| MG1655Δ*dnaK*:: *MXAN_3016* | pTrc99a- *MXAN_3016* plasmid transformed into MG1655Δ*dnaK* | This study |
| MG1655Δ*dnaK*:: *MXAN_3192* | pTrc99a- *MXAN_3192* plasmid transformed into MG1655ΔdnaK | This study |
| MG1655Δ*dnaK*:: *MXAN_5323* | pTrc99a- *MXAN_5323* plasmid transformed into MG1655Δ*dnaK* | This study |
| MG1655Δ*dnaK*:: *sglK* | pTrc99a- *MXAN_6671*plasmid transformed into MG1655Δ*dnaK* | This study |
| MG1655Δ*dnaK*:: *MXAN_7025* | pTrc99a- *MXAN_7025* plasmid transformed into MG1655Δ*dnaK* | This study |
| MG1655Δ*dnaK*:: *B0014* | pTrc99a-*b0014* plasmid transformed into MG1655Δ*dnaK* | This study |
| MG1655Δ*dnaK* | MG1655 Δ*b0014*(*dnaK*) | This study |
| Plasmids | | |
| pBJ113 | Gene replacement vector with KG cassette; Km^r^ | Laboratory collection |
| pBJ- *MXAN_2747* | Upstream and downstream homologous arms of DK1622 *MXAN_2747* and inserted | This study |
| pBJ- *MXAN_3016* | Upstream and downstream homologous arms of DK1622 *MXAN_3016 and inserted* | This study |
| pBJ- *MXAN_3192* | Upstream and downstream homologous arms of DK1622 *MXAN_3192*and inserted | This study |
| pBJ- *MXAN_5323* | Upstream and downstream homologous arms of DK1622 *MXAN_5323 and inserted* | This study |
| pBJ- *MXAN_6671* | Upstream and downstream homologous arms of DK1622 *MXAN_6671 and inserted* | This study |
| pBJ- *MXAN_7025* | Upstream and downstream homologous arms of DK1622 *MXAN_7025and inserted* | This study |
| pSWU30 | Site-specific integration vector with *Mx8 attB* integration site; Tet^r^ | Laboratory collection |
| pSWU30- *MXAN_2747* | *MXAN_2747* with 630bp *pilA* promotor sequence and inserted into XbaI/EcoRI of pSWU30, Tet^r^ | This study |
| pSWU30- *MXAN_3016* | *MXAN_3016* with 630bp *pilA* promotor sequence and inserted into XbaI/EcoRI of pSWU30, Tet^r^ | This study |
| pSWU30- *MXAN_3192* | *MXAN_3192* with 630bp *pilA* promotor sequence and inserted into XbaI/EcoRI of pSWU30, Tet^r^ | This study |
| pSWU30- *MXAN_5323* | *MXAN_5323* with 630bp *pilA* promotor sequence and inserted into XbaI/EcoRI of pSWU30, Tet^r^ | This study |
| pSWU30- *MXAN_6671* | *MXAN_6671* with 630bp *pilA* promotor sequence and inserted into XbaI/EcoRI of pSWU30, Tet^r^ | This study |
| pSWU30- *MXAN_7025* | *MXAN_7025* with 630bp *pilA* promotor sequence and inserted into XbaI/EcoRI of pSWU30, Tet^r^ | This study |
| pTrc99a | Expression vector, Amp^r^, trc promotor | Laboratory collection |
| pTrc99a- *MXAN_2747* | *MXAN_2747* insertion into pTrc99a | This study |
| pTrc99a- *MXAN_3016* | *MXAN_3016* insertion into pTrc99a | This study |
| pTrc99a- *MXAN_3192* | *MXAN_3192* insertion into pTrc99a | This study |
| pTrc99a- *MXAN_5323* | *MXAN_5323* insertion into pTrc99a | This study |
| pTrc99a- *MXAN_6671* | *MXAN_6671* insertion into pTrc99a | This study |
| pTrc99a- *MXAN_7025* | *MXAN_7025* insertion into pTrc99a | This study |
