# Supplementary material for: Bioinformatic and Functional Characterization of Hsp70s in Myxococcus xanthus
Source: mSphere. 2021 May 19;6(3):e00305-21. doi: 10.1128/mSphere.00305-21 (PMC8265645; doi:10.1128/mSphere.00305-21)
Supplement: TABLE S3 [file msphere.00305-21-st003.docx]

**Table S3.** List of primers used in this study.

| Primer | Sequence (5-3)* | Use |
| --- | --- | --- |
| *MXAN_2747*-KO-UF | TCGACTCTAGAGGATCCCCCGTGCAGTACAAGGGG | Amplification of upstream homologous arm for deletion of *MXAN_2747* |
| *MXAN_2747*-KO-UR | GGTCGAGTCGCGGCCTCCGTCTCCGGTGAG |  |
| *MXAN_2747*-KO-DF | GAGTGAGCGCCAGGGGCACCTCCTGCTCACCATT | Amplification of downstream homologous arm for deletion of *MXAN_2747* |
| *MXAN_2747*-KO-DR | TCGAGCTCGGTACCCCTGCTCCGGTGGC |  |
| *MXAN_3016*-KO-UF | TCGAGCTCGGTACCCACCTGCTGACGCCAG | Amplification of upstream homologous arm for deletion of *MXAN_3016* |
| *MXAN_3016*-KO-UR | GCGCTGGCTGCTCATGGGGAACCGTACTTA |  |
| *MXAN_3016*-KO-DF | TAAGTACGGTTCCCCATGAGCAGCCAGCGC | Amplification of downstream homologous arm for deletion of *MXAN_3016* |
| *MXAN_3016*-KO-DR | GTCGACTCTAGAGGATCCCCTTGAGCCAGATGATGTCG |  |
| *MXAN_3192*-KO-UF | AGCTCGGTACCCTGCCGCTGAGGA | Amplification of upstream homologous arm for deletion of *MXAN_3192* |
| *MXAN_3192*-KO-UR | CTCACCGCTTCCGAAGGTTCCTATCCTCCG |  |
| *MXAN_3192*-KO-DF | CGGAGGATAGGAACCTTCGGAAGCGGTGAG | Amplification of downstream homologous arm for deletion of *MXAN_3192* |
| *MXAN_3192*-KO-DR | CTCTAGAGGATCCCCATCAGCGTCATGCCCA |  |
| *MXAN_5323*-KO-UF | TCGAGCTCGGTACCCTTCCTCACCCGGCA | Amplification of upstream homologous arm for deletion of *MXAN_5323* |
| *MXAN_5323*-KO-UR | AGCATTCCTTCCAGTCACGCCTCCGATTGAC |  |
| *MXAN_5323*-KO-DF | GTCAATCGGAGGCGTGACTGGAAGGAATGCT | Amplification of downstream homologous arm for deletion of *MXAN_5323* |
| *MXAN_5323*-KO-DR | CTCTAGAGGATCCCCGGGACGTGTCCGCTA |  |
| *MXAN_6671*-KO-UF | GGCGATTGCGAGCGTGTTGGTTACTGACTC | Amplification of upstream homologous arm for deletion of *dnaK5* |
| *MXAN_6671*-KO-UR | CTCTAGAGGATCCCCAGGACGAGGTGACCG |  |
| *MXAN_6671*-KO-DF | TCGAGCTCGGTACCCCAGCTCCAGGCTGC | Amplification of downstream homologous arm for deletion of *dnaK5* |
| *MXAN_6671*-KO-DR | GAGTCAGTAACCAACACGCTCGCAATCGCC |  |
| *MXAN_7025*-KO-UF | GGATGGACGCGGGGGGCCGAGAGGAC | Amplification of upstream homologous arm for deletion of *MXAN_7025* |
| *MXAN_7025*-KO-UR | CTCTAGAGGATCCCCTTGGTTGCCCGGCGA |  |
| *MXAN_7025*-KO-DF | GGATGGACGCGGGGGGCCGAGAGGAC | Amplification of downstream homologous arm for deletion of *MXAN_7025* |
| *MXAN_7025*-KO-DR | CTCTAGAGGATCCCCTTGGTTGCCCGGCGA |  |
| PpilA-up | GCTCTAGACTGGCGAACTACTTCCTGTC | PCR amplification of pilA promotor for ligation with pswu30 |
| PpilA-down | CGGGATCCGGGGGTCCTCAGAGAA |  |
| *MXAN_2747*-OE-F | TCGACTCTAGAGGATCCCCCGTGCAGTACAAGGGG | PCR amplification of *MXAN_2747* for ligation with pSWU30 and pilA promotor |
| *MXAN_2747*-OE-R | GGTCGAGTCGCGGCCTCCGTCTCCGGTGAG |  |
| *MXAN_3016*-OE-F | TCTCTGAGGACCCCCATGGCTGAGCCCGAA | PCR amplification of *MXAN_3016* for ligation with pSWU30 and pilA promotor |
| *MXAN_3016*-OE-R | CCGGGGATCCTCTAGCTACCGCCCCGTGC |  |
| *MXAN_3192*-OE-F | TTCTCTGAGGACCCCCGTGGGCAAGATTATCGG | PCR amplification of *MXAN_3192* for ligation with pSWU30 and pilA promotor |
| *MXAN_3192*-OE-R | CCGGGGATCCTCTAGCTAAGACTGGCGGAA |  |
| *MXAN_5323*-OE-F | TCTCTGAGGACCCCCATGCGAATCGTTGGC | PCR amplification of *MXAN_5323* for ligation with pSWU30 and pilA promotor |
| *MXAN_5323*-OE-R | CCGGGGATCCTCTAGCTACGACAGCCGCAG |  |
| *MXAN_6671*-OE-F | TCTCTGAGGACCCCCATGGGCAAGGTGATT | PCR amplification of *MXAN_6671* for ligation with pSWU30 and pilA promotor |
| *MXAN_6671*-OE-R | CCGGGGATCCTCTAGTCAGCTCGCCTGGCC |  |
| *MXAN_7025*-OE-F | TCTCTGAGGACCCCCATGCATGACCTCGTC | PCR amplification of *MXAN_7025* for ligation with pSWU30 and pilA promotor |
| *MXAN_7025*-OE-R | CCGGGGATCCTCTAGTCAGGCGCGAGGCTC |  |
| *MXAN_2747*-OT-F | CACAGGAAACAGACCATGGCGGACGACATCGCAATC | PCR amplification of *MXAN_2747* for ligation with pTrc99a |
| *MXAN_2747*-OT-R | GCTGAAAATCTTCTCTTACTGCGCCGCGGCGGGCA |  |
| *MXAN_3016*-OT-F | CACAGGAAACAGACCATGGCTGAGCCCGAACCCCTCA | PCR amplification of *MXAN_3016* for ligation with pTrc99a |
| *MXAN_3016*-OT-R | GCTGAAAATCTTCTCCTACCGCCCCGTGCCGCCGG |  |
| *MXAN_3192*-OT-F | CACAGGAAACAGACCGTGGGCAAGATTATC | PCR amplification of *MXAN_3192* for ligation with pTrc99a |
| *MXAN_3192*-OT-R | GCTGAAAATCTTCTCCTAAGACTGGCGGAAC |  |
| *MXAN_5323*-OT-F | CACAGGAAACAGACCATGCGAATCGTTGGCATCGACCTG | PCR amplification of *MXAN_5323* for ligation with pTrc99a |
| *MXAN_5323*-OT-R | GCTGAAAATCTTCTCCTACGACAGCCGCAGACCGGCG |  |
| *MXAN_6671*-OT-F | CACAGGAAACAGACCATGGGCAAGGTGATT | PCR amplification of *MXAN_6671* for ligation with pTrc99a |
| *MXAN_6671*-OT-R | GCTGAAAATCTTCTCTCAGCTCGCCTGGCC |  |
| *MXAN_7025*-OT-F | CACAGGAAACAGACCATGCATGACCTCGTC | PCR amplification of *MXAN_7025* for ligation with pTrc99a |
| *MXAN_7025*-OT-R | GCTGAAAATCTTCTCTCAGGCGCGAGGCTC |  |
| *B0014*-OT-F | CACAGGAAACAGACCATGGGTAAAATAATT | PCR amplification of *dnaK* of *E.coli* for ligation with pTrc99a |
| *B0014*-OT-R | GCTGAAAATCTTCTCTTATTTTTTGTCTTTGAC |  |
| pTrc99a-up | GGTCTGTTTCCTGTGTGAAA | PCR amplification of pTrc99a for ligation with mutiple *Hsp70* genes |
| pTrc99a-down | GAGAAGATTTTCAGCCTGATAC |  |
